# Recruitment of PVT1 Enhances YTHDC1-Mediated m6A Modification of IL-33 in Hyperoxia-Induced Lung Injury During Bronchopulmonary Dysplasia

**DOI:** 10.1007/s10753-023-01923-1

**Published:** 2023-11-02

**Authors:** Tianping Bao, Xiangye Liu, Jian Hu, Mengmeng Ma, Jingyan Li, Linxia Cao, Bingrui Yu, Huaiping Cheng, Sai Zhao, Zhaofang Tian

**Affiliations:** 1 Department of Neonatology, The Affiliated Huaian No.1 People’s Hospital of Nanjing Medical University, No. 1 Western Huanghe Road, Huai’an, 223300 Jiangsu China; 2 Department of Pediatrics, The Affiliated Huaian No.1 People’s Hospital of Nanjing Medical University, Huai’an, Jiangsu, China

**Keywords:** bronchopulmonary dysplasia, m6A methylation, RNA binding protein, alveolar epithelial cells, PVT1, IL-33

## Abstract

Bronchopulmonary dysplasia (BPD) is a chronic lung disease that specifically affects preterm infants. Oxygen therapy administered to treat BPD can lead to hyperoxia-induced lung injury, characterized by apoptosis of lung alveolar epithelial cells. Our epitranscriptomic microarray analysis of normal mice lungs and hyperoxia-stimulated mice lungs revealed elevated RNA expression levels of IL-33, as well as increased m6A RNA methylation levels of IL-33 and PVT1 in the hyperoxia-stimulated lungs. This study aimed to investigate the role of the PVT1/IL-33 axis in BPD. A mouse model of BPD was established through hyperoxia induction, and lung histological changes were assessed by hematoxylin–eosin staining. Parameters such as radial alveolar count and mean chord length were measured to assess lung function. Mouse and human lung alveolar epithelial cells (MLE12 and A549, respectively) were stimulated with hyperoxia to create an *in vitro* BPD model. Cell apoptosis was detected using Western blotting and flow cytometry analysis. Our results demonstrated that silencing PVT1 suppressed apoptosis in MLE12 and A549 cells and improved lung function in hyperoxia-stimulated lungs. Additionally, IL-33 reversed the effects of PVT1 both *in vivo* and *in vitro*. Through online bioinformatics analysis and RNA-binding protein immunoprecipitation assays, YTHDC1 was identified as a RNA-binding protein (RBP) for both PVT1 and IL-33. We found that PVT1 positively regulated IL-33 expression by recruiting YTHDC1 to mediate m6A modification of IL-33. In conclusion, silencing PVT1 demonstrated beneficial effects in alleviating BPD by facilitating YTHDC1-mediated m6A modification of IL-33. Inhibition of the PVT1/IL-33 axis to suppress apoptosis in lung alveolar epithelial cells may hold promise as a therapeutic approach for managing hyperoxia-induced lung injury in BPD.

## Introduction

Bronchopulmonary dysplasia (BPD) is a chronic lung disease that affects preterm infants requiring respiratory support and oxygen therapy after birth [1]. Oxygen toxicity, inflammatory injury, and genetic predisposition are risk factors associated with this disease [2–4]. Studies have highlighted the involvement of an imbalance between pro-inflammatory and anti-inflammatory mechanisms in the pathogenesis of BPD [5, 6]. Hyperoxia leads to the excessive production of reactive oxygen species, damages lung epithelial cells, increases lung permeability, and promotes the levels of inflammatory cytokines [7]. Notably, immature alveolar epithelial cells, particularly type II alveolar epithelial cells, are primary targets of hyperoxia-induced lung injury in premature infants [8]. Extensive research has demonstrated the role of alveolar epithelial cell apoptosis in hyperoxia-stimulated lungs of neonatal rats [8–10]. Despite considerable progress in the clinical management of BPD, the incidence of this disease remains relatively stable, highlighting the need for novel therapeutic targets [11].

Hyperoxia-induced methylation has been observed in a rodent model of BPD [12], and DNA methylation has been detected in autopsy lung samples from preterm infants with BPD [13]. Among the various internal mRNA modifications, N6-methyladenosine (m6A) is the most common and abundant, recognized by m6A-selective reader proteins of the YTH family [14]. YTH domain-containing 1 (YTHDC1), a nuclear m6A reader, facilitates the transport of m6A methylated mRNA from the nucleus to the cytoplasm [15]. The involvement of YTHDC1-mediated m6A RNA methylation has been investigated in numerous diseases, including various human tumors [16] and osteosarcoma [17]. Both YTHDF1 mRNA expression and m6A-RNA methylation levels increased in the mouse model of acute respiratory distress syndrome [18]. However, there have been no previous studies on m6A RNA methylation in BPD nor its reader YTHDC1.

Interleukin-33 (IL-33), a member of the IL-1 cytokine family, is constitutively expressed in healthy mucosal tissues and other organs, with increased expression under inflammatory conditions [19]. IL-33 plays a role in chronic lung inflammation [20–22], inducing the formation of neutrophil extracellular traps and degrading fibronectin in a mouse model of BPD [23]. Knockdown of IL-33 has been shown to exert protective effects against hyperoxia-induced lung injury, which is attributed to reduced polarization and proliferation of alveolar macrophages [24]. Elevated cord blood IL-33 levels have been observed in severe BPD (3.91 ± 1.22 pg/mL) than moderate BPD (2.82 ± 0.74) group in premature infants [25]. In a neonatal IL-33 transgenic mouse model, enlarged alveolar spaces resembling BPD have been observed [19]. Moreover, IL-33 is associated with alveolar epithelial cell injury [26–28], underscoring its crucial role in BPD.

Plasmacytoma variant translocation gene 1 (PVT1) is a long non-coding RNA (lncRNA) whose expression is upregulated by inflammatory stimuli [29–31]. PVT1 has been implicated in promoting airway inflammation during asthma [32] and recently identified as a regulator of the asthmatic phenotype in human airway smooth muscle [33]. High expression levels of PVT1 have been detected in peripheral blood samples from patients with chronic obstructive pulmonary disease, indicating its potential as a biomarker for this condition [34]. However, the exact role of PVT1 in BPD remains unclear.

In our study, we performed Arraystar Mouse m6A-mRNA&lncRNA Epitranscriptomic microarray analysis on normal lungs (n = 3, the control group) and hyperoxia-stimulated lungs (n = 3, the BPD group). We identified significantly higher RNA expression levels of IL-33 and increased m6A RNA methylation levels of IL-33 and PVT1 (with the highest significance) in the BPD group compared to the control group. Therefore, we proposed a hypothesis that PVT1 and IL-33 may be involved in the occurrence and development of BPD.Our investigation aimed to elucidate the role of PVT1 in the apoptosis of hyperoxia-stimulated alveolar epithelial cells and a mouse model of BPD. We also examined the molecular interaction between PVT1 and IL-33, along with their relationship with m6A RNA methylation.

## Materials and Methods

### Cell Culture

Mouse alveolar epithelial cell line MLE12 (#IM-M015) and human alveolar epithelial cell line A549 (#IM-H113) were purchased from IMMOCELL (Xiamen, China). MLE12 cells were cultured in DMEM/F12 (94%) + fetal bovine serum (2%) + GlutaMAX-1 glutamine (1%) + HEPES 1 M Buffer solution (1%) + penicillin–streptomycin (1%) + ITS (Insulin + transferrin + Selenium) (1%) + hydrocortisone (10 nM) + 10 nM (estradiol). A549 cells were cultured in 90% DMEM + 10% fetal bovine serum + penicillin–streptomycin. To construct the *in vitro* model of BPD, MLE12, and A549 cells were exposed to 100% oxygen for 16 h [35].

### Vector Construction and Transfection

Short hairpin-RNAS (sh-RNAs) targeting PVT1 (sh-PVT1), YTHDC1 (sh-YTHDC1), IL-33 (sh-IL-33), and scrambled shRNAs as negative control (sh-NC) were synthesized by GenePharma (Shanghai, China). The coding region sequences of YTHDC1 were inserted into pcDNA3.1 vector (GenePharma) to construct the overexpression vector, and the empty vector was used as negative control (NC). All vectors were transfected into MLE12 and A549 cells using lipofectamine 2000 (ThermoFisher Scientific) at room temperature based on the manufacturer’s instructions.

### Quantitative Real-Time Polymerase Chain Reaction (qRT-PCR)

Total RNA was extracted from MLE12 and A549 cells, as well as hyperoxia-stimulated lungs, using TRIzol Reagent (#15596026), followed by reverse transcription using a RevertAid RT kit (#K1691). Subsequently, q-PCR was performed using SYBR™ Green PCR Master Mix (#4309155). All the reagents were purchased from ThermoFisher Scientific, and all procedures were performed according to the instructions of supplier. The results were calculated by the 2^−ΔΔCt^ method, and each sample was run in triplicate.

### Western Blotting

Proteins were extracted from MLE12 and A549 cells, as well as hyperoxia-stimulated lungs. The protein samples were mixed with the loading buffer, separated by 10% SDS-PAGE at 100 V for 3 h, and transferred to PVDF membranes at 100 V for 50 min. After blocking in 5% skimmed milk for 3 h at room temperature, the membranes were incubated overnight at 4 °C with primary antibodies, including rabbit monoclonal anti-Bax (ab32503; 1:2000), rabbit monoclonal anti-Bcl-2 (ab182858; 1:2000), or rabbit monoclonal anti-GAPDH (ab181602; 1:10000). The membranes were then washed with TBST three times and incubated with goat anti-rabbit secondary antibody anti-IgG (ab6721; 1:10000) for 2 h. Protein bands were visualized using enhanced chemiluminescence reagents (ThermoFisher Scientific). All antibodies were purchased from Abcam (Shanghai, China). The Image Lab 5.0 software (Bio-Rad) was used to analyze the densitometry values of Bax and Bcl-2, which were standardized relative to GAPDH.

### Cell Counting Kit-8

Cells were seeded in 96-well plates at a density of 5 × 10^3^ cells/well and cultured for 24 h. CCK8 solution (Beyotime) was added to each well, and the plates were further incubated at 37 °C for 3 h. Cell viability, indicated by the optical density of each well, was measured using a microplate reader (Bio-Rad) at 450 nm.

### Flow Cytometry Analysis

An Annexin V-FITC/PI Apoptosis Detection Kit (#A211-01, Nanjing, China) was used according to the manufacturer’s instructions. Transfected MLE-12 and A549 cells were seeded into 6-well plates at a concentration of 1 × 10^5^ cells/well, washed with cold PBS, and then resuspended in annexin-binding buffer. Next, cells were incubated with 5 µL of Annexin V-FITC and 10 µL of PI for 10 min at room temperature in the dark. Apoptotic cells were detected with the CytoFLEX flow cytometer and analyzed using the CytExpert software (Beckman). Cells in Q3 were defined as apoptotic cells.

### RNA-Binding Protein Immunoprecipitation (RIP)

A RNA Immunoprecipitation Kit (#P0102, Geneseed, Guangzhou, China) was used following the manufacturer’s instructions. A/G magnetic beads were incubated with anti-IgG (#ab133470, Abcam), anti-YTHDC1 (#ab264375, Abcam), and anti-m6A (#ab208577, Abcam) and then added to the cell lysates. After elution, RNA was isolated from the complexes and analyzed by q-PCR, as mentioned above, to detect the enrichment of PVT1 or IL-33.

### RNA Pull-Down Assay

Biotinylated RNA was treated with structure buffer to induce secondary structure formation, denatured by heating and ice-bath for ion, and then incubated with BeyoMag™ Streptavidin Magnetic Beads (#P2151, Beyotime) for 2 h at 4 °C. Cell lysates of MLE12 and A549 cells were divided into four groups (Input, Bio-NC, Bio-IL-33-wt, and Bio-IL-33-m6A mut) and incubated with the bead-probe complex at overnight 4 °C. After extracting total proteins from pull-down products, Western blot was conducted to detect the enrichment of YTHDC1 as mentioned above.

### Luciferase Reporter Assay

A Dual Luciferase Reporter Gene Assay Kit (RG027, Beyotime, Shanghai, China) was used according to the manufacturer’s instructions. The full sequence of IL-33 was subcloned into the pGL3 vector to construct the pGL3-IL-33 (m6A-Wt) vector. IL-33 with mutated m6A binding site was inserted into the pGL3 vector to create the pGL3-IL-33 (m6A-Mut) vector. The empty pGL3vector served as NC. After co-transfection of the reporter vectors mentioned above with pcDNA3.1-YTHDC1 or pcDNA3.1 into MLE12 and A549 cells, the luciferase reporter activity was determined using the Dual-Luciferase^®^ Reporter Assay System (E1910, Promega), with Renilla luciferase used as an internal reference.

### Establishment of Hyperoxia-Induced BPD Mouse Model

All animal procedures were performed in accordance with the Guide for the Care and Use of Laboratory Animals published by the National Institute of Health, under the approval of the Ethics Committee of The Affiliated Huaian No.1 People's Hospital of Nanjing Medical University. Neonatal mice were placed in a sealed polypropylene cage and exposed to continuous oxygen at a flow rate of 5 L/min with an inspired fraction of O_2_ > 95% [36, 37], as measured by an oxygen analyzer, from postnatal day 1 to 7. The temperature was maintained between 22–27 °C, with humidity levels between 50 and 70%. The cage contained soda lime to absorb CO_2_ and color-changing silica gel to absorb water. To maintain lung inflation, the trachea of the mice was intubated, exsanguination was performed, the chest was opened, and the lungs were subsequently extracted.

### Intervention of the Hyperoxia-Treated Mice

Before hyperoxia treatment, mice in the BPD/PVT1 KO group were intratracheally instilled with 5 μL adenovirus vector expressing sh-PVT1 at a titer of 1 × 10^9^ pfu/100 μL. Mice in the BPD/PVT1 KO + IL-33 group were intratracheally instilled with 5 μL adenovirus vector expressing sh-PVT1 and 5 μL adenovirus vector expressing IL-33. The adenovirus vectors were synthesized by GenePharma Co. Ltd. (Shanghai, China).

### Hematoxylin-Eosin (HE) Staining

Hyperoxia-stimulated lung tissues were fixed with 4% paraformaldehyde overnight at 4 °C, embedded in paraffin, and sectioned into 5 μm slices. Subsequently, the sections were deparaffinized, hydrated, and stained with HE. A light microscope (Nikon, Tokyo, Japan) was used to observe the pathological changes of lung tissues under five randomly selected fields. The radial alveolar count was calculated along a vertical line from the center of the respiratory bronchiole to the distal pleura [36]. The alveolar chord length was measured using Image-Pro Plus 6.0 software.

### Statistical Analysis

All data were presented as mean ± standard deviation and analyzed using GraphPad Prism9. Difference comparisons among multiple groups were performed using one-way ANOVA, followed by Tukey's post hoc test for pairwise comparisons. The *t*-test was used for comparing differences between two groups. *P* < 0.05 indicated statistical significance. For *in vitro* assays, three biological repeats and three technical repeats were performed. For *in vivo* assays, either three or six biological repeats and three technical repeats were conducted.

## Results

### Elevated m6A Methylation Levels of IL-33 and PVT1 in BPD

**Fig. 1:**
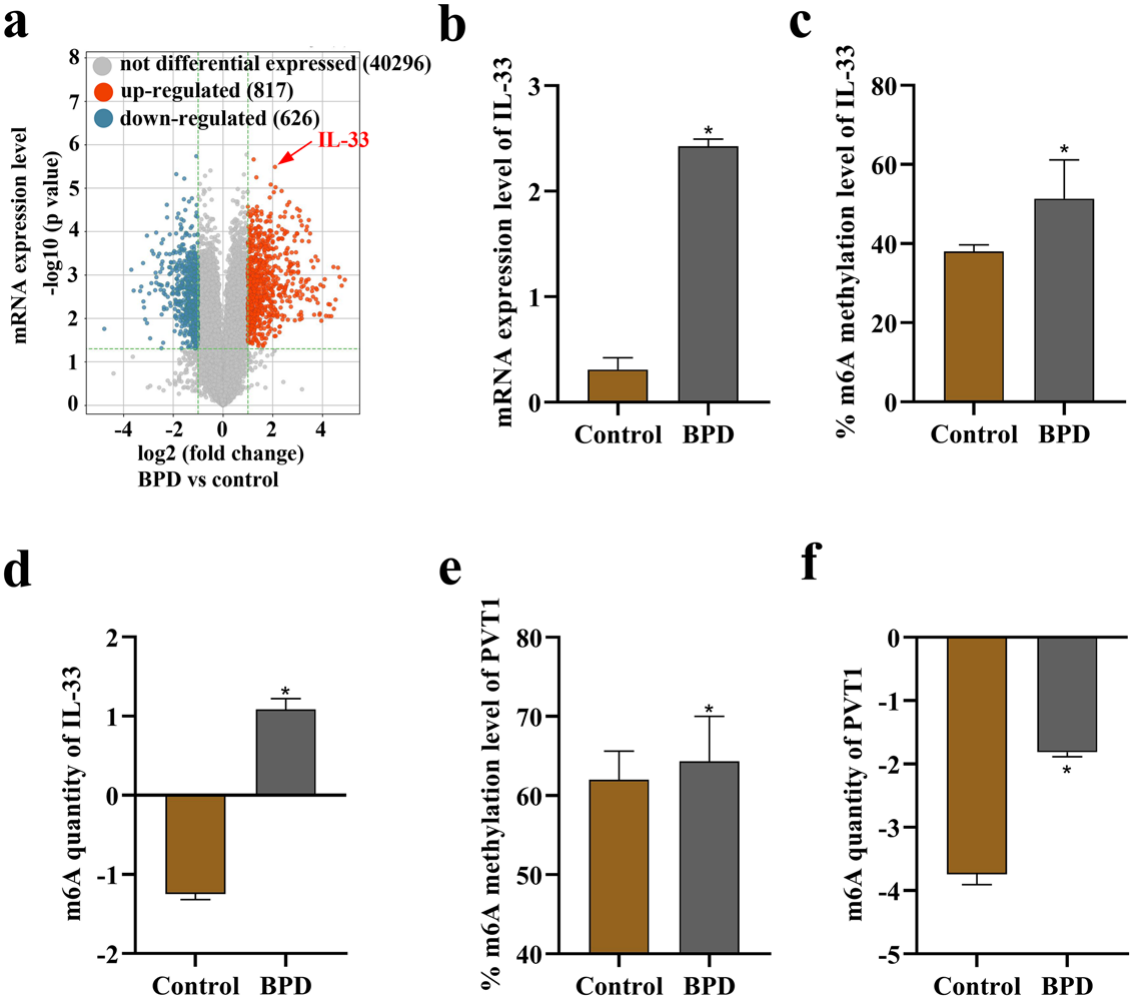
High m6A methylation levels of IL-33 and PVT1 in BPD. **a** Volcano map illustrating the differentially expressed mRNAs between the control group and the BPD group. The red arrow indicates IL-33. **b** Comparison of RNA expression levels of IL-33 between the control group and the BPD group. **c**, **d** Analysis of m6A methylation levels and m6A quantity of IL-33 between the control group and the BPD group. **e**, **f** Evaluation of m6A methylation levels and m6A quantity of PVT1 between the control group and the BPD group. *p < 0.05.

Sequence analysis revealed 817 upregulated mRNAs and 626 downregulated mRNAs in the BPD group compared to the control group, as depicted in Fig. 1a. In the BPD group, IL-33 exhibited higher RNA expression levels (Fig. 1b), m6A methylation level (Fig. 1c), and m6A abundance (Fig. 1d). Similarly, Fig. 1e, f demonstrated that PVT1 displayed higher RNA expression levels in the BPD group. These findings suggest the involvement of m6A modification in IL-33 and PVT1 in BPD.

### Silencing of PVT1 and IL-33 Reduces Hyperoxia-Stimulated Cell Death in MLE12 and A549 Cells

**Fig. 2:**
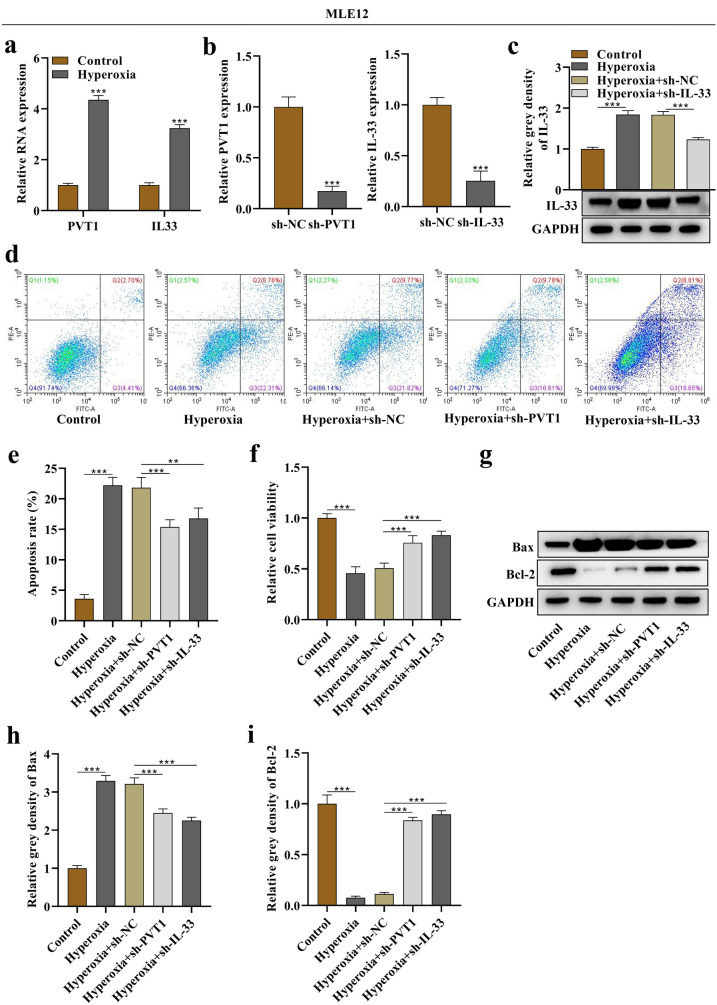
Silencing of PVT1 and IL-33 reduced death of hyperoxia-stimulated MLE12 cells. **a** Relative RNA expression of PVT1 and IL-33 in basal or hyperoxia-stimulated MLE12 cells. **b** Assessment of silencing efficiency of PVT1 and IL-33 in MLE12 cells by PCR. **c** Western blotting band of IL-33 protein in MLE12 cells and its corresponding quantitative grey density (relative to GAPDH). **d** Flow cytometry analysis of apoptosis in MLE12 cells of the control, hyperoxia, hyperoxia + sh-NC, hyperoxia + sh-PVT1, and hyperoxia + sh-IL-33 groups. **e** Quantitative data of apoptosis rate. **f** Evaluation of cell viability. **g** Western blotting bands of Bax and Bcl-2 proteins in MLE12 cells. **h** Quantitative grey density of Bax. **i** Quantitative grey density of Bcl-2. **p < 0.01, ***p < 0.001.

Hyperoxia led to a 4.35-fold upregulation of PVT1 RNA expression and a 3.23-fold upregulation of IL-33 RNA expression in MLE12 cells (Fig. 2a). Successful silencing of PVT1 and IL-33 was achieved using the respective shRNAs (Fig. 2b). Figure 2c demonstrated that IL-33 protein expression was enhanced by hyperoxia but decreased upon sh-IL-33 treatment. MLE12 cell apoptosis rate increased under hyperoxia and was further reduced by sh-PVT1 or sh-IL-33 (Fig. 2d, e). sh-PVT1 or sh-IL-33 counteracted the suppressive effects of hyperoxia on MLE12 cell viability (Fig. 2f). Moreover, the expression levels of the apoptosis markers Bax (proapoptotic) and Bcl-2 (antiapoptotic) were assessed, revealing that Bax expression increased under hyperoxia and decreased upon sh-PVT1 or sh-IL-33 treatment, while Bcl-2 exhibited opposite trends (Fig. 2g–i).

**Fig. 3:**
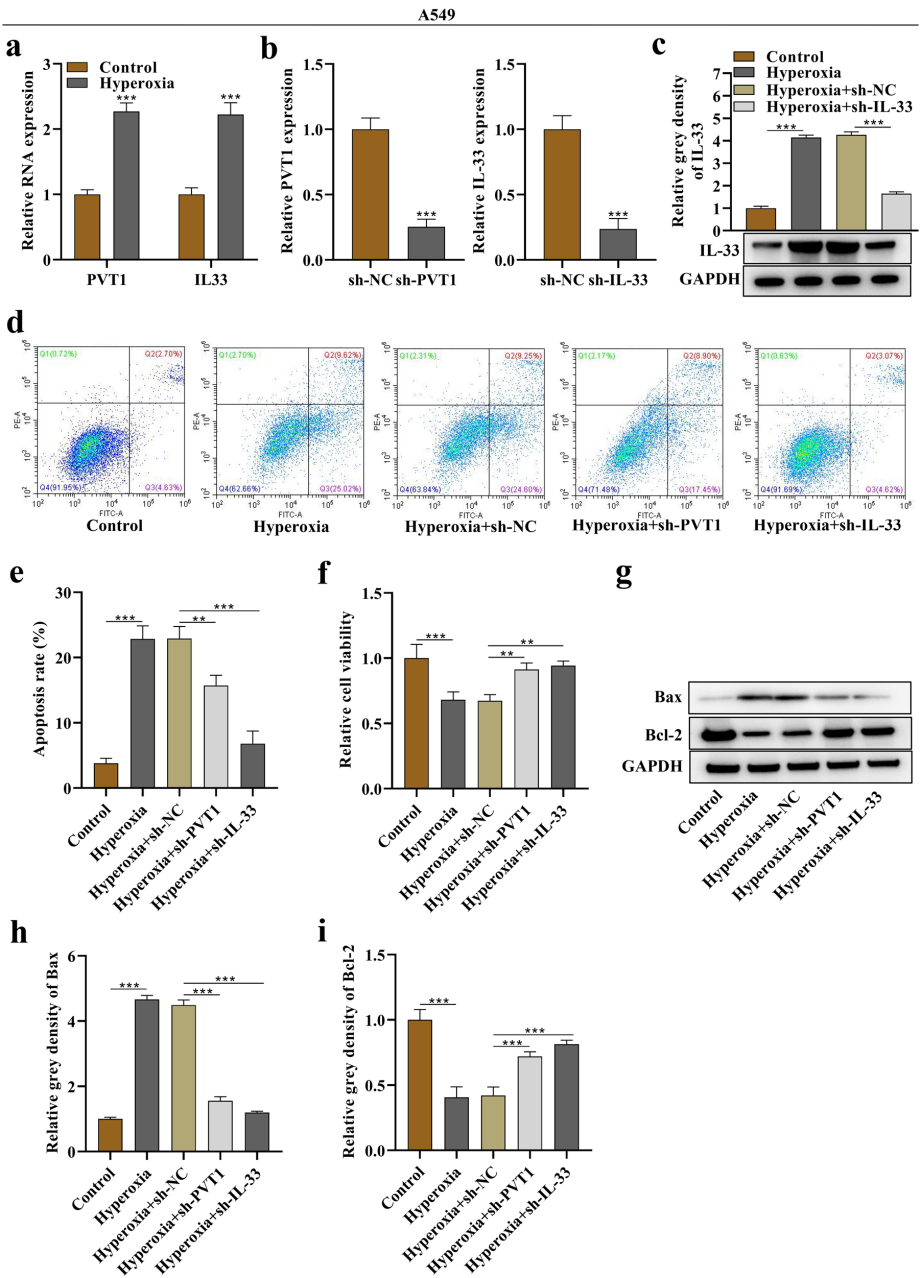
Silencing of PVT1 and IL-33 decreased death of hyperoxia-stimulated A549 cells. **a** Relative RNA expression of PVT1 and IL-33 in basal or hyperoxia-stimulated A549 cells. **b** PCR analysis of the silencing efficiency of PVT1 and IL-33 in A549 cells. **c** Western blotting band of IL-33 protein in A549 cells and its corresponding quantitative grey density (relative to GAPDH). **d** Flow cytometry analysis of apoptosis in A549 cells of the control, hyperoxia, hyperoxia + sh-NC, hyperoxia + sh-PVT1, and hyperoxia + sh-IL-33 groups. **e** Quantitative data of apoptosis rate. **f** Evaluation of cell viability using a CCK-8 kit **g** Western blotting bands of Bax and Bcl-2 proteins in A549 cells. **h**, **i** Quantitative grey densities of Bax and Bcl-2. **p < 0.01, ***p < 0.001.

A human alveolar epithelial cell A549 was also stimulated with hyperoxia to investigate the roles of PVT1 and IL-33 in alveolar epithelial cell death. The results were consistent with those observed in MLE12 cells, demonstrating that hyperoxia upregulated PVT1 and IL-33 expression, while sh-PVT1 or sh-IL-33 rescued hyperoxia-induced A549 cell apoptosis and reversed the suppressive effects of hyperoxia on A549 cell viability (Fig. 3a–i).

### PVT1 and YTHDC1 Positively Regulate IL-33 Expression

**Fig. 4:**
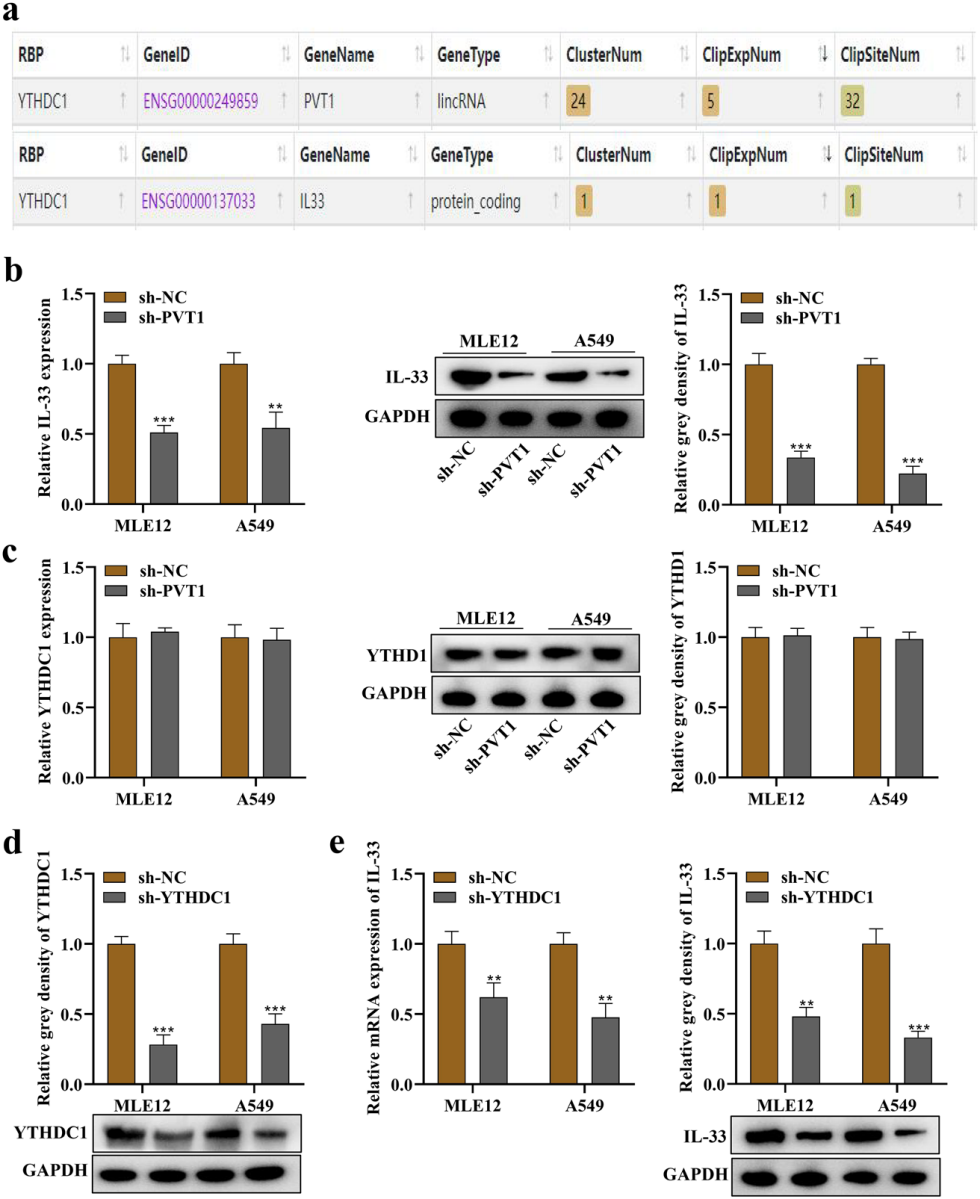
IL-33 expression was positively regulated by PVT1 and YTHDC1. **a** The ENCORI database revealed the binding of both PVT1 and IL-33 to YTHDC1 protein. **b** Silencing PVT1 resulted in reduced RNA and protein expression of IL-33 and in MLE12 and A549 cells, as indicated by PCR and Western blot analysis. **c** Silencing PVT1 had no significant effect on the RNA and protein expression of YTHDC1 in MLE12 and A549 cells, as observed by PCR and western blot analysis. **d** Western blot analysis of YTHDC1 protein in MLE12 and A549 cells by transfection of sh-YTHDC1. **e** Silencing YTHDC1 led to a decrease in IL-33 RNA and protein expression, as indicated by PCR and Western blot analysis. **p < 0.01, ***p < 0.001.

Bioinformatics analysis revealed that YTHDC1 is a RBP that interacts with both PVT1 and YTHDC1 (Fig. 4a). Cells treated with sh-PVT1 exhibited a significant decrease in IL-33 RNA and protein expression (Fig. 4b). PVT1 had no influence on YTHDC1 expression, either at the RNA or protein level (Fig. 4c). To silence endogenous YTHDC1 expression in MLE12 and A549 cells, sh-YTHDC1 was designed. The efficiency of sh-YTHDC1 was confirmed by the reduced grey density of YTHDC1 observed through Western blotting analysis (Fig. 4d). YTHDC1 suppression led to a decrease in IL-33 RNA and protein expression (Fig. 4e).

### PVT1 Modulates IL-33 Through YTHDC1-mediated M6A Modification

**Fig. 5:**
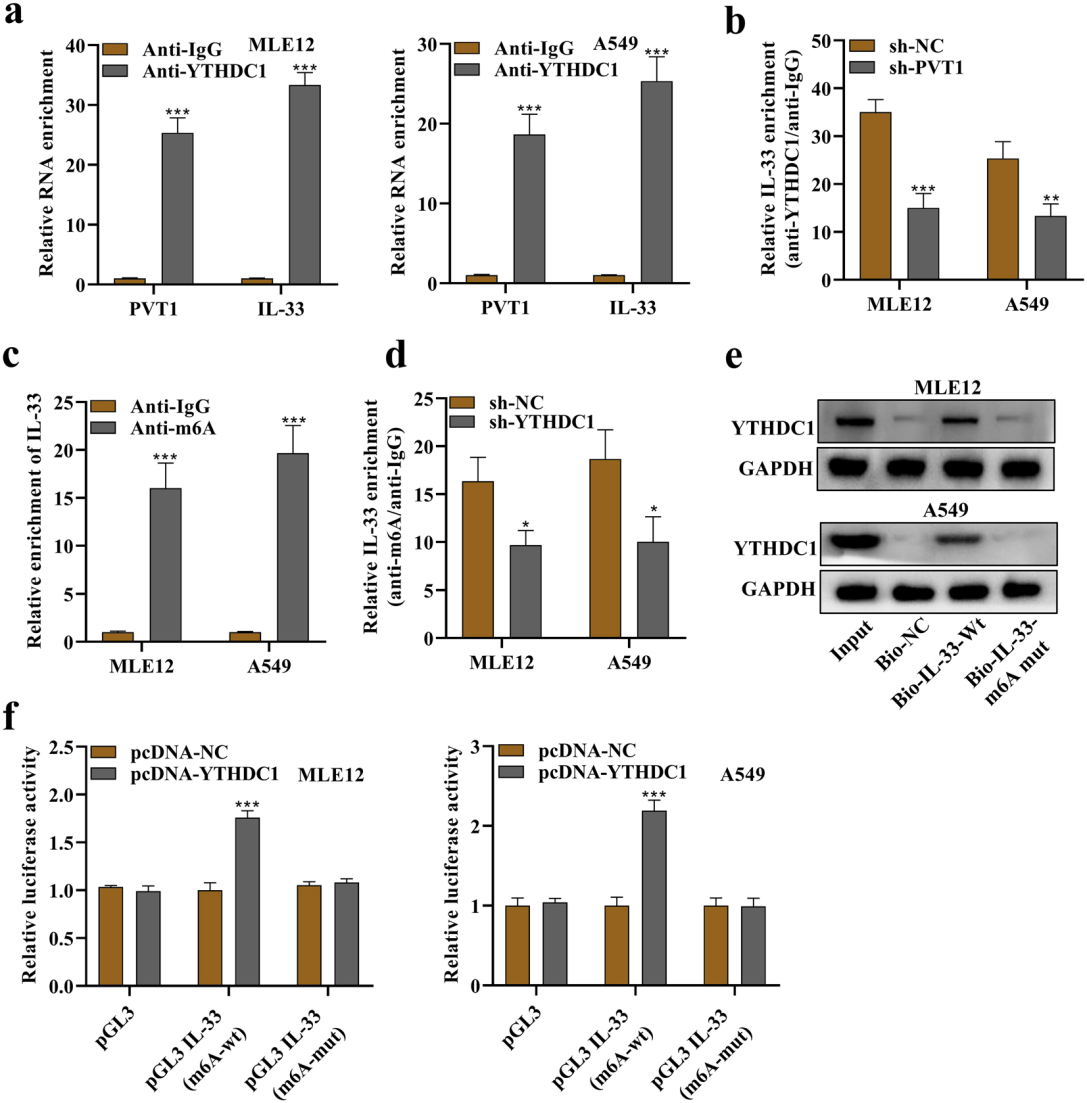
PVT1 modulated IL-33 by YTHDC1-mediated m6A modification. **a** A RIP assay using YTHDC1 antibody demonstrated the binding of both PVT1 and IL-33 to the YTHDC1 protein in MLE12 and A549 cells. **b** After silencing PVT1, the binding of IL-33 and YTHDC1 protein was further assessed using a RIP assay. **c** A RIP assay using m6A antibody was performed to demonstrate the binding of IL-33 and m6A. **d** After silencing YTHDC1, the binding of IL-33 and m6A protein was further assessed by RIP assay. **e** RNA pull-down assay followed by Western blotting was performed to reveal YTHDC1 protein expression that was pulled down by bio-IL-33-Wt or bio-IL-33-m6A Mut. **f** A luciferase reporter assay revealed the luciferase activity of MLE12 and A549 cells by cotransfection of pcDNA-YTHDC1 and empty pGL3, or pGL3 IL-33 (m6A Wt), or pGL3 IL-33 (m6A Mut). *p < 0.05, **p < 0.01, ***p < 0.001.

The interaction between YTHDC1 protein and PVT1/IL-33 in MLE12 and A549 cells was confirmed using a RIP assay (Fig. 5a). sh-PVT1 treatment reduced the binding of YTHDC1 protein and IL-33 by more than 50% (Fig. 5b). We also observed a strong affinity between IL-33 and m6A (Fig. 5c), which was decreased by sh-YTHDC1 (Fig. 5d). Subsequently, a RNA pull-down assay was conducted, which confirmed that the binding between YTHDC1 protein and IL-33 was m6A dependent (Fig. 5e). As shown in Fig. 5f, pcDNA-YTHDC1 enhanced the luciferase activity of pGL3-IL-33 (m6A wt), while no significant changes were observed with pGL3-IL-33 (m6A mut), indicating that YTHDC1 mediates the m6A methylation of IL-33.

### Rescue Effects of IL-33 in *In Vivo* and *In Vitro* Models of BPD in the Absence of PVT1

**Fig. 6:**
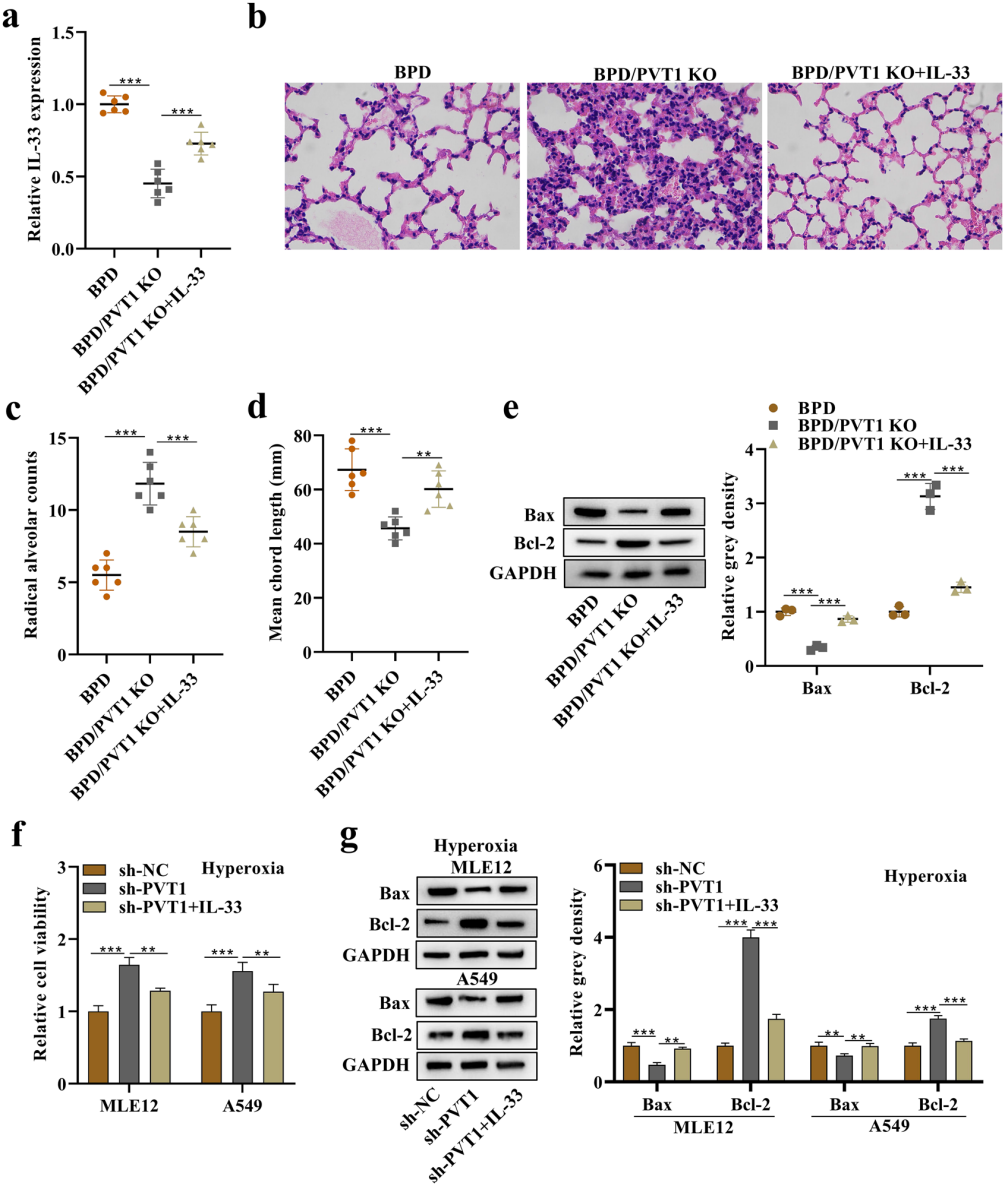
Rescue effects of IL-33 in the *in vivo* and *in vitro* models of BPD in the absence of PVT1. **a** Expression of IL-33 in hyperoxia-stimulated lungs of the BPD, BPD/PVT1 KO, and BPD/PVT1 KO + IL-33 groups. **b** H&E staining of hyperoxia-stimulated lungs. **c** Quantification of radial alveoli. **d** Mean chord length (mm). **e** Western blot analysis of Bax and Bcl-2 proteins in hyperoxia-stimulated lungs. **f** Viability of hyperoxia-treated MLE12 and A549 cells was detected by a CCK-8 kit. **g** Western blotting bands of Bax and Bcl-2 in hyperoxia-treated MLE12 and A549 cells. **p < 0.01, ***p < 0.001.

The rescue influences of IL-33 on PVT1 were investigated in hyperoxia-stimulated mice or alveolar epithelial cells. Lung IL-33 expression was reduced by PVT1 knockdown in mice (Fig. 6a). PVT1 knockdown improved the pathological features of hyperoxia-stimulated lungs, as evidenced by an increase in alveolar numbers and a decrease in alveolar size. Intervention with IL-33 significantly rescued these effects (Fig. 6b). Radial alveolar count and mean chord length are important indicators of lung development. Radial alveolar count increased upon PVT1 knockdown and decreased with IL-33 treatment (Fig. 6c), while mean chord length was decreased by PVT1 knockdown and increased by IL-33 treatment (Fig. 6d). Figure 6e demonstrated that PVT1 knockdown reduced Bax protein expression and enhanced Bcl-2 protein expression in hyperoxia-stimulated lungs, and these effects were partially restored by IL-33. In hyperoxia-stimulated MLE12 and A549 cells, PVT1 knockdown increased cell viability (Fig. 6f) and decreased apoptosis (Fig. 6g), while IL-33 partially reversed these effects.

## Discussion

Previous studies have highlighted the crucial roles of lncRNAs in the progression and treatment of BPD [38–41]. In this study, we have made an innovative discovery by demonstrating the significant upregulation of PVT1 in hyperoxia-stimulated lung alveolar epithelial cells through PCR analysis. Additionally, using epitranscriptomic microarray analysis, we have identified high levels of m6A RNA methylation in PVT1 in BPD mice. Our findings indicate that PVT1 knockdown reduces apoptosis and enhances viability in hyperoxia-stimulated lung alveolar epithelial cells. Furthermore, silencing PVT1 reduces alveolar size, increases radial alveolar count, and decreases mean chord length in hyperoxia-stimulated mice. These findings suggest that targeting PVT1 and suppressing its expression could have a beneficial effect in the treatment of BPD.

IL-33 exhibits dual functions depending on its cellular location. While the full-length IL-33 is found within the nucleus of cells, the mature IL-33 acts as an extracellular cytokine and is released when cells detect inflammatory signals or undergo necrosis [42–44]. The conversion from full-length IL-33 to mature IL-33 is facilitated by neutrophil-derived proteases [42]. Previous research by Larouche *et al*. revealed lower DNA methylation levels of IL33 in bronchial epithelial cells of asthmatic individuals compared to controls [45]. In our study, we observed higher levels of m6A RNA methylation and mRNA expression of IL33 in BPD mice using epitranscriptomic microarray analysis. We also observed upregulation of IL33 mRNA expression in hyperoxia-stimulated lung alveolar epithelial cells. Considering that hyperoxia can induce methylation in BPD [12], it can be inferred that the upregulation of IL-33 mRNA in hyperoxia-induced BPD is dependent on its m6A RNA methylation. Furthermore, our study demonstrated that silencing IL33 has a negative effect on the apoptosis of hyperoxia-stimulated lung alveolar epithelial cells, consistent with previous studies highlighting the pro-apoptotic role of IL-33 [46, 47]. The results of our rescue assays further indicated that IL-33 partially reverses the effects of PVT1 silencing in both *in vivo* and *in vitro* models of BPD.

Moreover, our study suggests that the interaction between PVT1 and IL-33 is mediated by YTHDC1-mediated m6A methylation. We confirmed that YTHDC1 is a RBP that binds to both PVT1 and IL-33 in lung alveolar epithelial cells. Both PVT1 and YTHDC1 positively regulate IL-33 mRNA expression. Upon silencing YTHDC1, the binding of m6A to IL-33 is inhibited. PVT1 positively regulates IL-33 expression by recruiting YTHDC1 to mediate m6A modification on IL-33, without affecting YTHDC1 expression. As an m6A reader, YTHDC1 can stabilize or destabilize mRNAs by functioning as a RBP [48, 49], and it has been implicated as a risk factor in lung cancer [50] and acute respiratory distress syndrome [18]. However, the direct role of YTHDC1 in BPD remains unclear, which is a limitation of our study.

Moving forward, our research will focus on elucidating the source of the m6A modification in PVT1 in BPD. Building upon the studies conducted by Shen *et al.* [51] and Chen *et al.* [52], which indicates that ALKBH5 promotes the stability of PVT1 through m6A modification, our future investigations will validate the interaction between ALKBH5 and PVT1 and explore the potential roles of ALKBH5 in BPD. Moreover, hyperoxia-induced oxidative stress in lung alveolar epithelial cells leads to apoptosis [7]. PVT1 has been identified as a regulator of oxidative stress [53], and there is a bi-directional cause-and-effect relationship between oxidative stress and IL-33 [54, 55]. Therefore, our upcoming studies will also investigate the relationship between PVT1/IL-33 and oxidative stress in BPD.

## Conclusions

In conclusion, our study presents a novel finding by revealing high expression levels of m6A RNA methylation and RNA expression of PVT1/IL-33 in BPD. We demonstrate that PVT1 upregulates IL-33 expression by recruiting YTHDC1, an m6A reader protein, to mediate the m6A modification of IL-33. Silencing PVT1 suppresses lung alveolar cell apoptosis and improves lung architecture in a hyperoxia-stimulated mouse model of BPD, dependent on IL-33.

## Data Availability

The original contributions presented in the study are included in the article, and further inquiries can be directed to the corresponding author.
